# Optical Tissue Clearing and Small-Molecule Labeling of Paraffin-Embedded Breast Cancer and Axillary Lymph Node Human Tissue Samples

**DOI:** 10.21769/BioProtoc.5790

**Published:** 2026-08-20

**Authors:** Lisa D.J. Schiffelers, Uma Pisarović, Albert Bitorina, Sven Hildebrand, Britt Walravens, Thiemo J.A. van Nijnatten, Loes F.S. Kooreman, Anna Schueth

**Affiliations:** 1 Department of Genetics & Cell Biology, Maastricht University, Maastricht, The Netherlands; 2 Department of Cognitive Neuroscience, Maastricht University, Maastricht, The Netherlands; 3 Department of Radiology and Nuclear Medicine, Maastricht University Medical Centre, Maastricht, the Netherlands; 4 GROW Research Institute for Oncology and Reproduction, Maastricht University, Maastricht, The Netherlands; 5 Dutch Expert Center for Screening (LRCB), Nijmegen, The Netherlands; 6 Department of Pathology, Maastricht University Medical Centre, Maastricht, The Netherlands; 7 PNUTRIM Research Institute for Nutrition and Translational Research in Metabolism, Maastricht University, Maastricht, The Netherlands

**Keywords:** Optical tissue clearing, Breast cancer, Lymph nodes, Human cancer tissue samples, FFPE tissue samples

## Abstract

Breast cancer is the most frequently diagnosed cancer in women, representing approximately 25% of all cancers in women worldwide. Both breast cancer research and histopathological diagnostics mainly show a two-dimensional planar view of the three-dimensional breast cancerous architecture. Recently, the application of optical tissue clearing, together with 3D microscopy, has been applied to visualize the complexity of whole tumor samples. Preliminary studies on whole-organ mouse mammary glands and tissues from human breast cancer patients subjected to optical tissue clearing and volumetric imaging have enabled the detection of previously unrecognized spatial cellular interactions and structural features within intact breast tissue. There is currently no standardized clearing workflow for breast and lymph node tissues. In this protocol, we optimized and validated the MASH (multiscale architectonic staining of human cortex) immunolabeling-enabled three-dimensional imaging of solvent-cleared organs (iDISCO)-like clearing and labeling pipeline for the investigation of formalin-fixed and paraffin-embedded (FFPE) breast tissue and lymph nodes obtained from breast cancer patients. This illustrates the application of the protocol in a new biological and clinical context, as human breast and lymph node tissues differ substantially from brain tissues in their composition, architecture, and optical properties. Whole FFPE tissue blocks are deparaffinized in liquid paraffin and xylene, bleached through methanol dehydration and a subsequent hydrogen peroxide incubation, and stained with a diverse set of small molecule dyes. As a next step, the tissues are delipidated and subjected to refractive index matching with ethyl cinnamate to reach optimal tissue transparency. Importantly, the applied dehydration and delipidation nicely preserve the morphology of the tissue, and the shrinkage is minimal. This allows reliable 3D imaging of large tissue samples within a timeframe of 10 days, providing clinicians and biomedical researchers with a more holistic view of the FFPE tissue sample and its spatial organization.

Key features

• Optimized, low-cost approach for optical tissue clearing and labeling of whole human breast and lymph node FFPE tissue samples ≥30 × 20 × 3 mm.

• Prepared tissue samples can be assessed by means of advanced 3D volume microscope modalities, such as twophoton microscopy (TPM) or light-sheet fluorescence microscopy (LSFM).

• The clearing protocol is compatible with a variety of small-molecule dyes, such as neutral red (cell body), eosin Y (cytoplasm, collagen), and methyl green (nucleus).

## Graphical overview


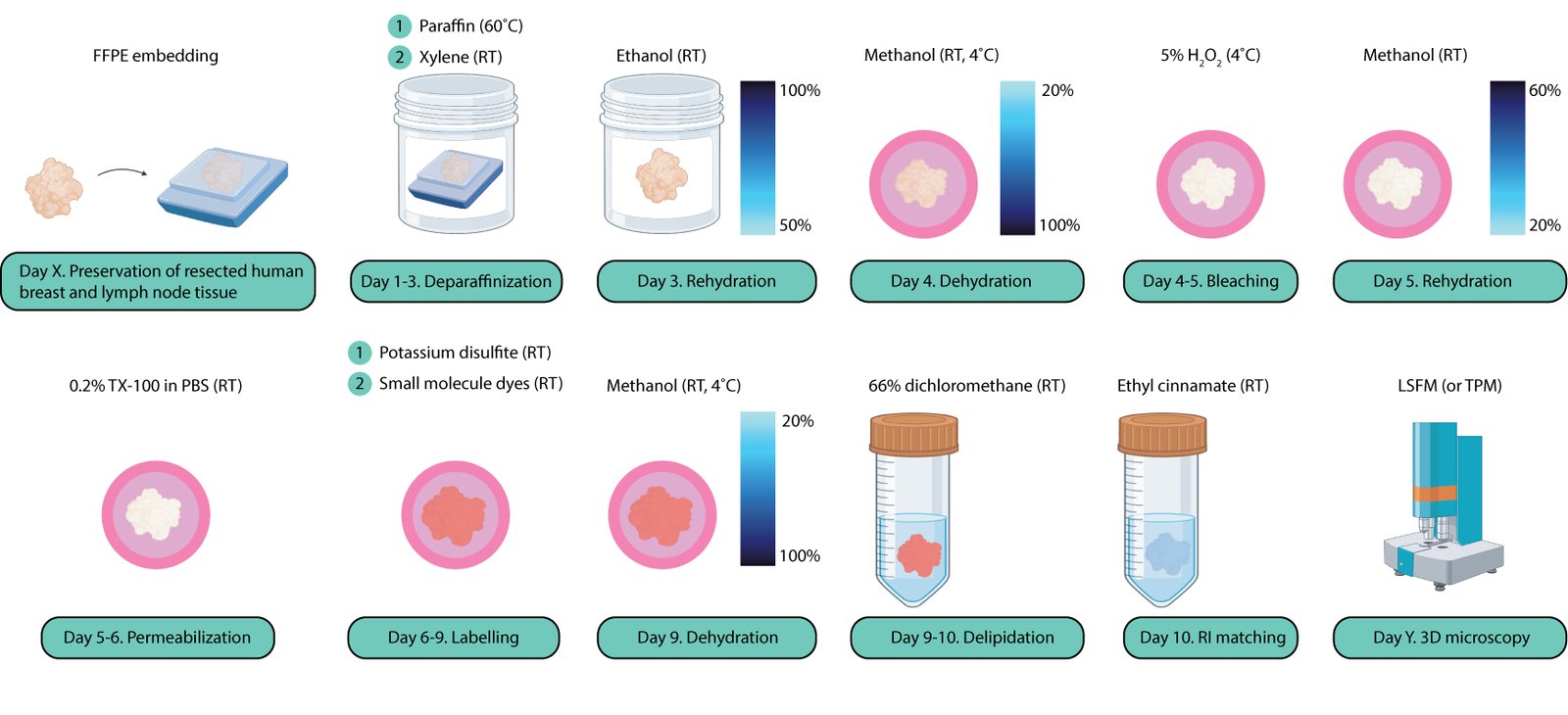


**Schematic overview of the optical tissue clearing and small-molecule labeling steps of human breast cancer and lymph node tissue samples.** This illustration was created using Adobe Illustrator and https://BioRender.com.

## Background

With approximately 2.3 million new cases and over 670,000 deaths annually, breast cancer remains the leading type of cancer in women worldwide [1]. There are several distinct subtypes, with invasive breast carcinoma of no special type (NST) and invasive lobular carcinoma (ILC) being the most common [2]. To date,20T **i20T**n the field of histopathology, clinicians and pathologists have relied on histological examinations of 3–5 μm-thin formalin-fixed and paraffin-embedded (FFPE) 2D tissue slices subjected to hematoxylin and eosin (H&E) staining. Despite recent advances in the level of early detection and precision medicine, patient numbers are increasing; therefore, new methodologies to improve diagnostics and patient therapy are essential [1]. At the same time, most breast cancer research is primarily focused on two-dimensional models, which only enable a limited, planar representation of the large-scale 3D breast cancer tissue architecture. As a result, key characteristics of large-scale human cancer tissue samples, such as the tumor volume, the high-resolution 3D tissue architecture, and the spatial organization of distinct cell populations, have remained largely unexplored due to the lack of suitable tools and methodologies.

Recent advances in optical tissue clearing, paired with light-sheet fluorescence microscopy (LSFM), are beginning to overcome the limitations of conventional slide-based breast cancer studies, enabling three-dimensional volume assessment of breast tissue samples [3]. Optical tissue clearing renders several millimeter thick tissues samples transparent, which circumvents scattering during image acquisition, resulting in deep microscopic imaging [4]. Barner et al. demonstrated that cleared, FFPE breast cancer lymph nodes imaged with LSFM offer a non-destructive, 3D alternative to traditional 2D histology with classic imaging modalities [5]. Cleared-tissue LSFM has also been applied to examine the terminal ductal lobular unit (TDLU) architecture and cell phenotypes in healthy breast tissue, contributing to our understanding of normal breast histology and its alterations in disease [6]. Despite these advances, workflows that combine optical tissue clearing and non-destructive 3D imaging of large FFPE human breast cancer tissues and their associated axillary lymph nodes remain underdeveloped. Robust and reproducible protocols for such tissues, both malignant and non-malignant, are still limited.

Here, we adapted and refined the MASH (multiscale architectonic staining of human cortex) immunolabeling-enabled three-dimensional imaging of solvent-cleared organs (iDISCO)-like optical tissue clearing and labeling protocol for human breast and lymph node tissues [7–9]. Our protocol demonstrates optical tissue clearing of complete, unsectioned FFPE tissue blocks of both breast and lymph nodes from breast cancer patients with dimensions of at least 30 × 20 × 3 mm. As the first step describes the deparaffinization of FFPE material, the protocol is applicable and suitable for archival tissue samples and their retrospective analyses. The combination of small molecule dyes with this optical tissue clearing protocol allows the specific visualization of subcellular compartments such as the nucleus, the lysosomes, and the cytoplasm. This allows for the establishment of a robust and broadly applicable 3D imaging workflow, which is less expensive and time-consuming than the use of immunohistochemistry. Antibody-based labeling has already been successfully combined with other iDISCO-based clearing protocols, and the list of verified antibodies is growing [10]. However, it has to be noted that other antibodies still have to be tested and verified; hence, here we focus on a universally transferable method. The cleared and labeled tissue specimen can eventually be examined with various microscope modalities, such as multiphoton or light-sheet fluorescence microscopy. This way, the potential for 3D analysis is harnessed and combined with LSFM advantages, including low levels of photobleaching, high imaging speed, and an exceptional signal-to-noise ratio [11]. In the future, this technique has the potential to aid in the investigation of immune cell infiltration, intratumoral heterogeneity, vascularization, and the spatial relations between malignant, stromal, and immune cell populations for both research and clinical investigations [12].

## Materials and reagents

**Biological materials**

1. Human breast cancer samples of surgical specimens of the primary tumor site within the breast, preserved as FFPE tissue blocks, obtained from the Pathology Department of Maastricht University Medical Center+

2. Human lymph node samples of surgical specimens from breast cancer patients undergoing axillary surgery, preserved as FFPE tissue blocks, obtained from the Pathology Department of Maastricht University Medical Center+

*Note: After the clearing process, tissue samples are stored in ethyl cinnamate in Falcon tubes at room temperature (RT) until imaging or in the cold room at 4 °C for longer storage.*

**Reagents**

1. Liquid paraffin (histological grade) (Carl Roth, catalog number: 9190.1)

2. Xylene (Laboratorium Discounter, catalog number: XY834.1)

3. Ethanol, absolute (100%) (Carl Roth, catalog number: t913.2)

4. Methanol, absolute (100%) (Carl Roth, catalog number: 4627.4)

5. Milli-Q water

6. Phosphate-buffered saline (PBS), 0.1 M (Carl Roth, catalog number: 9143.2)

7. Thymol (Carl Roth, catalog number: 5391.1)

8. TritonP^TM^P X-100 (octylphenol ethoxylate) (Sigma-Aldrich, catalog number: X100)

9. 30% hydrogen peroxide (HR_2_ROR_2_R) (Carl Roth, catalog number: cp26.1)

10. Potassium disulfite (KR_2_RSR_2_ROR_5_R) (Carl Roth, catalog number: 7995.1)

11. Disodium hydrogen phosphate dihydrate (NaR_2_RHPOR_4_R·2HR_2_RO) (Carl Roth, catalog number: 27ke.1)

12. Citric acid monohydrate (CR_6_RHR_8_ROR_7_R·HR_2_RO) (Carl Roth, catalog number: 3958.2)

13. Neutral Red (NR) (Carl Roth, catalog number: T122.3)

14. Methyl Green (MG) (Sigma-Aldrich, catalog number: M8884)

15. Eosin Y (Morphisto, catalog number: 12199.00100)

16. 4′,6-Diamidino-2-phenylindole (DAPI) stock (1 mg/mL) (Carl Roth, catalog number: 6843.3)

17. Dichloromethane (DCM) (Carl Roth, catalog number: 8424.2)

18. Ethyl cinnamate (ECi) (Sigma-Aldrich, catalog number: 112372)

19. Chloroform (trichloromethane) (Carl Roth, catalog number: 6340.1)

**Solutions**

1. 50% (v/v) ethanol (see Recipes)

2. 70% (v/v) ethanol (see Recipes)

3. 20% (v/v) methanol (see Recipes)

4. 40% (v/v) methanol (see Recipes)

5. 50% (v/v) methanol (see Recipes)

6. 60% (v/v) methanol (see Recipes)

7. 80% (v/v) methanol (see Recipes)

8. 5% (v/v) hydrogen peroxide (HR_2_ROR_2_R) (see Recipes)

9. 50% (w/w) potassium disulfite solution (see Recipes)

10. McIlvaine buffer (phosphate-citrate buffer), pH 4.0 (see Recipes)

11. NR stock solution, 0.1% (w/v) (see Recipes)

12. MG stock solution, 4% (w/v) (see Recipes)

13. DAPI stock solution, 1 mg/mL (v/v) (see Recipes)

14. Dichloromethane:methanol (DCM) (2:1, v/v) (see Recipes)

15. 0.1 M PBS + 0.2% (v/v) Triton X-100 (see Recipes)

**Recipes**

See General note 1.

**1. 50% (v/v) ethanol**

**Table:** 

Reagent	Final concentration	Quantity or volume
Ethanol, absolute	50% (v/v)	50 mL
Milli-Q water	/	50 mL
Total	/	100 mL

First, measure absolute ethanol (100%) using a graduated cylinder and dilute with Milli-Q water to the final volume. Then, mix thoroughly by gentle agitation. Prepare at the laboratory bench, away from ignition sources, due to ethanol flammability. Store absolute ethanol and all ethanol dilutions at RT in tightly closed, clearly labeled containers in a flammable-liquids storage cabinet, according to institutional safety regulations. Keep containers away from heat sources and open flames.

**2. 70% (v/v) ethanol**

**Table:** 

Reagent	Final concentration	Quantity or volume
Ethanol, absolute	70% (v/v)	70 mL
Milli-Q water	/	30 mL
Total	/	100 mL

**3. 20% (v/v) methanol**

**Table:** 

Reagent	Final concentration	Quantity or volume
Methanol, absolute	20% (v/v)	20 mL
Milli-Q water	/	80 mL
Total	/	100 mL

First, measure absolute methanol (100%) using a graduated cylinder and dilute with Milli-Q water to the final volume. Then, mix thoroughly by gentle agitation. Prepare in a chemical fume hood due to methanol toxicity and volatility. Store absolute methanol and all methanol dilutions at RT in tightly closed, clearly labeled containers in a flammable-liquids storage cabinet, according to institutional safety regulations. Due to methanol toxicity and volatility, ensure containers are clearly labeled and minimize opening time during use.

**4. 40% (v/v) methanol**

**Table:** 

Reagent	Final concentration	Quantity or volume
Methanol, absolute	40% (v/v)	40 mL
Milli-Q water	/	60 mL
Total	/	100 mL

**5. 50% (v/v) methanol**

**Table:** 

Reagent	Final concentration	Quantity or volume
Methanol, absolute	50% (v/v)	50 mL
Milli-Q water	/	50 mL
Total	/	100 mL

**6. 60% (v/v) methanol**

**Table:** 

Reagent	Final concentration	Quantity or volume
Methanol, absolute	60% (v/v)	60 mL
Milli-Q water	/	40 mL (≈40 g)
Total	/	100 mL

**7. 80% (v/v) methanol**

**Table:** 

Reagent	Final concentration	Quantity or volume
Methanol, absolute	80% (v/v)	80 mL
Milli-Q water	/	20 mL
Total	/	100 mL

**8. 5% (v/v) H**R**_2_** R**O**R**_2_**

**Table:** 

Reagent	Final concentration	Quantity or volume
HR_2_ROR_2_R stock	30% (v/v)	16.7 mL
Methanol	/	83.3 mL
Total	5% (v/v)	100 mL

To prepare a 5% (v/v) hydrogen peroxide solution, measure the required volume of 30% stock using a volumetric pipette and dilute in pre-cooled methanol. Add hydrogen peroxide slowly to the methanol while gently mixing by agitation. Prepare the solution in a chemical fume hood due to the strong oxidative properties of hydrogen peroxide and the potential release of oxygen gas. Protect the solution from light at all times. Prepare the diluted hydrogen peroxide solution immediately prior to use. It is not advised to store diluted solutions, as hydrogen peroxide rapidly decomposes upon dilution and light exposure. Store 30% (v/v) hydrogen peroxide stock at 4 °C in its original, vented, light-protected container, according to the manufacturer’s instructions.

**9. 50% (w/v) potassium disulfite solution**

**Table:** 

Reagent	Final concentration	Quantity or volume
KR_2_RSR_2_ROR_5_R stock	50% (w/w)	50 g
Milli-Q water	/	50 mL
Total	/	100 mL

First, weigh KR_2_RSR_2_ROR_5_R using an analytical balance and then add Milli-Q water until just before the final volume. Stir with a magnetic stir while heating the solution up to approximately 60–70 °C. When dissolved, add the remaining Milli-Q to the final volume. Please note that the solution is oversaturated and the salt will precipitate again over time. Avoid inhalation of dust. Store at RT in a tightly sealed glass container. Directly before use, it is advised to stir the solution and heat it up on a magnet stirrer to dissolve the precipitate. This is followed by filtering the clear dissolved solution.

**10. McIlvaine buffer (phosphate-citrate buffer), pH 4.0**

Prepare 0.2 M NaR_2_RHPOR_4_R and 0.1 M citric acid stock solutions separately by weighing salts on an analytical balance and dissolving them in Milli-Q water to the required volumes by mixing on a magnetic stir. Combine the two solutions in the specified ratio to achieve pH 4.0 and mix thoroughly. Prepare at the laboratory bench. Store the buffer at 4 °C.

See General note 2.

**Step 1. 0.2 M Na**R**_2_** R**HPO**R**_4_** R **stock solution**

**Table:** 

Reagent	Final concentration	Quantity or volume
NaR_2_RHPOR_4_R·2HR_2_RO	0.2 M	35.447 g
Milli-Q water	/	1,000 mL
Total	/	1,000 mL

**Step 2. 0.1 M C**R**_6_** R**H**R**_8_** R**O**R**_7_** R**·H**R**_2_** R**O stock solution**

**Table:** 

Reagent	Final concentration	Quantity or volume
CR_6_RHR_8_ROR_7_R·HR_2_RO	0.1 M	21.017 g
Milli-Q water	/	1,000 mL
Total	/	1,000 mL

**Step 3. Final phosphate-citrate buffer**

**Table:** 

Reagent	Final concentration	Quantity or volume
0.2 M NaR_2_RHPOR_4_R solution	/	40 mL
0.1 M citric acid solution	/	60 mL
Total	/	100 mL

**11. NR stock solution, 0.1% (w/v)**

**Table:** 

Reagent	Final concentration	Quantity or volume
NR	0.1 mg/mL (0.1%)	1 mg
McIlvaine buffer (pH 4.0)	/	10 mL
Total	/	10 mL

First, prepare a 0.1% (0.1 mg/mL) stock solution by weighing NR using an analytical balance and dissolving it in McIlvaine buffer (pH 4.0). Mix gently until fully dissolved. Dilute the stock dilution 1:100 in McIlvaine buffer (pH 4.0) to obtain the final 0.001% working concentration. Prepare at the laboratory bench under reduced light conditions. Protect from light and store at 4 °C. Store NR powder at RT in a tightly closed container, protected from light and moisture. See General note 3.

**12. MG stock solution, 4% (w/v)**

**Table:** 

Reagent	Final concentration	Quantity or volume
MG	40 mg/mL (4%)	40 mg
McIlvaine buffer (pH 4.0)	/	1 mL
Total	/	1 mL

First, prepare a highly concentrated 4% MG aqueous stock solution according to [13]. Remove crystal violet impurities by extractions with chloroform in a separation funnel, thereby discarding the lower (violet) phase until the lower chloroform phase no longer contains the violet color. Dilute the stock solution 1:5,000 in McIlvaine buffer (pH 4.0). Prepare at the laboratory bench under reduced light conditions. Protect from light and store at 4 °C. Store MG powder at RT in a tightly sealed container, protected from light and humidity. See General note 3.

**13. DAPI stock solution, 1 mg/mL (v/v)**

**Table:** 

Reagent	Final concentration	Quantity or volume
DAPI	1 mg/mL	10 mg
McIlvaine buffer (pH 4.0)	/	10 mL
Total	/	10 mL

First, prepare a 1 mg/mL stock solution by weighing DAPI using an analytical balance and dissolving it in McIlvaine buffer (pH 4.0). Mix gently until fully dissolved and minimize light exposure. Store DAPI stock solutions at -20 °C, protected from light, according to the manufacturer’s instructions. Dilute the DAPI stock solution 1:1,000 in McIlvaine buffer (pH 4.0) to obtain the final 1 μg/mL working solution. Prepare at the laboratory bench immediately prior to use. See General note 3.

**14. DCM (2:1, v/v)**

**Table:** 

Reagent	Final concentration	Quantity or volume
DCM	66% (v/v)	66.7 mL
Methanol, absolute	33%	33.3 mL
Total	/	100 mL

Measure DCM and methanol using glass graduated cylinders and combine in a 2:1 (v/v) ratio. It is advised to prepare this strictly in a chemical fume hood due to the high volatility and toxicity of both dichloromethane and methanol. Stir until the inhomogeneities disappear. Store dichloromethane in tightly closed, chemically compatible containers at RT in a dedicated halogenated-solvent storage cabinet, separate from flammable solvents. Ensure containers are clearly labeled and kept away from heat sources. See General notes 4 and 5.

**15. 0.1 M PBS + 0.2% (v/v) Triton X-100**

**Table:** 

Reagent	Final concentration	Quantity or volume
Triton X-100	0.2% (v/v)	0.2 mL
PBS	0.1 M	99.8 mL
Total	/	100 mL

Prepare 0.1 M PBS using MilliQ water. Triton X-100 is highly viscous, which can complicate pipetting. To facilitate handling, the end of the pipette tip may be cut. Solutions containing Triton X-100 should not be shaken; instead, gently stir and allow the detergent to dissolve passively, which may take up to 1 h.

**Laboratory supplies**

1. Glass containers, 100, 250, and 500 mL

2. Glass Petri dish

3. Beakers, 50 and 200 mL

4. Erlenmeyer flask, 50 mL

5. Graduated cylinders, 25, 50, 100, 250, and 500 mL

6. 50 mL Falcon tubes

7. Six-well cell culture plates

8. Pipettes, 0.2, 1, and 5 mL, with compatible pipette tips

9. Forceps

10. Filter paper

11. Funnel

12. Millimeter paper

13. Nitrile gloves

## Equipment

1. Water bath, set to 60 °C (Gesellschaft für Labortechnik mbH, model: 1002)

2. Fume hood (Vinitex Laboratoriuminrichtingen B.V., model: L1K1-GR+51)

3. Chemical waste disposal system (institutional facility)

4. Orbital shaker (Polymax, model: 1040)

5. Cold room or refrigerated storage, 4 °C

6. Analytical balance (Mettler Toledo, model: PG2002-S DeltaRange)

7. Magnetic stirrer (Framo®-Gerätetechnik, model: M21/1)

8. Led-light pad (optional) (Crafts & Co, Karsten International, catalog number: 973)

9. Two-photon (2P) laser scanning microscope (Leica TCS SP5 MP, Leica Mikrosysteme Vertrieb GmbH)

10. UltraMicroscope II light-sheet microscope (La Vision Biotech, Miltenyi)

## Software and datasets

1. Leica Application Suite Advanced Fluorescence (Leica Microsystems), for Leica 2-photon microscopy data

2. Imaris Biplane 11.0, for 2-photon microscopy and LSFM 3D volume data

3. FIJI (open-source Bio-image analysis software)

## Procedure

*Note: See General notes 6 and 7.*

**A. Deparaffinization of human FFPE breast and lymph node samples**

1. Place each FFPE tissue sample in 50 mL of liquid paraffin in a glass container.

2. Incubate samples for 24–48 h in a water bath preheated to 60 °C until the paraffin is dissolved. The exact duration depends on paraffin thickness, so checking regularly is advised.

3. Transfer samples to a glass container containing 50 mL of xylene and incubate at RT on an orbital shaker (30 rpm) for 1 h.

4. Replace the xylene with fresh 50 mL of xylene and incubate for an additional 1 h at RT on a shaker (30 rpm).

5. Replace the xylene once more with fresh 50 mL of xylene and incubate overnight at RT on a shaker (30 rpm).

*See General note 8.*

6. Rehydrate samples by sequential incubation in 50 mL of ethanol solutions (see Recipes 1 and 2) prepared in distilled water, each for 1 h at RT on a shaker (30 rpm), using glass containers throughout, in the following order: 100% ethanol (twice), 70% ethanol, and 50% ethanol.

7. For overnight storage, incubate samples in 50 mL of 0.1 M PBS in a glass container at RT to ensure efficient and even equilibration on a shaker (30 rpm).

*See General note 9.*

**B. Clearing and labeling of deparaffinized human breast and lymph node samples**

**B1. Methanol pretreatment and bleaching**

1. Transfer samples to the six-well plates.

2. Dehydrate samples by sequential incubation in 8 mL of ascending methanol concentrations (see Recipes 3, 4, 6, and 7) prepared in distilled water, each for 1 h at RT on a shaker (30 rpm), as follows: 20% methanol, 40% methanol, 60% methanol, and 80% methanol.

3. Incubate samples in 8 mL of 100% methanol for 1 h at RT on a shaker (30 rpm) to maximize the dehydration step. Next, transfer the samples in fresh 100% methanol to the cold room for 1 h to stabilize the tissue before the oxidative bleaching (again, shake at 30 rpm).

4. Prepare a fresh 5% HR_2_ROR_2_R solution (see Recipe 8).

5. Bleach samples overnight at 4 °C on a shaker (30 rpm) in 8 mL per sample of 5% HR_2_ROR_2_R solution.

6. Allow samples to re-equilibrate to RT.

7. Rehydrate samples through descending methanol concentrations, each for 1 h at RT on a shaker (30 rpm), as follows: 60% methanol, 40% methanol, and 20% methanol.

8. Complete the methanol removal with a detergent-mediated 1-h incubation with 8 mL per sample of 0.1 M PBS containing 0.2% (v/v) Triton X-100 (pH 7.4) (see Recipe 15), followed by overnight incubation in a fresh solution, both at RT on a shaker (30 rpm).

**B2. Labeling with small molecule dyes**

1. Incubate samples in 8 mL of freshly filtered 50% (w/v) potassium disulfite solution (see Recipe 9) for 1 h at RT on a shaker (30 rpm).

*See General notes 10 and 11.*

2. Rinse samples five times briefly with distilled water.

3. Incubate samples in 8 mL of distilled water for 1 h at RT on a shaker (30 rpm).

4. Prepare the following staining solutions in phosphate-citrate buffer (McIlvaine buffer, pH 4.0) (see Recipe 10):

a. 0.001% Neutral Red → dilute the 0.1% stock solution 1:100 (see Recipe 11).

b. 0.00025% Methyl Green → dilute the 4% stock solution 1:5,000 (see Recipe 12).

c. 0.00025% eosin Y → dilute the commercial 0.5% stock solution 1:2,000.

d. 1 μg/mL DAPI → dilute the stock solution of 1 mg/mL 1:1,000 (see Recipe 13).

5. Incubate samples in 8 mL per sample of the selected dye solution for 4 days at RT on a shaker (30 rpm).

*See General note 12.*

6. Flip samples halfway through the incubation period to ensure uniform staining.

**B3. Clearing and refractive index matching with ECi**

1. Wash samples twice in 8 mL of McIlvaine buffer (pH 4.0) for 1 h each at RT on a shaker (30 rpm).

2. Dehydrate samples through ascending methanol concentrations prepared in distilled water, each for 1 h at RT on a shaker (30 rpm), as follows: 20% methanol, 50% methanol (see Recipe 5), 80% methanol, and 100% methanol (twice).

3. Transfer samples to 50 mL conical centrifuge tubes (Falcon tubes).

4. Incubate samples overnight in 33% methanol/66% DCM (v/v) (see Recipe 14) at RT on a shaker (30 rpm) for delipidation. Completely fill up the Falcon tube to reduce tissue browning through oxidation.

*See General notes 4 and 5.*

5. Remove residual methanol by incubating samples twice in 35 mL of 100% DCM, each for 1 h at RT sideways on a shaker (30 rpm) to facilitate optimal mixing.

*See General notes 4 and 5.*

6. Immerse the samples in ECi for refractive index matching and again completely fill the Falcon tube with ECi to reduce oxidation-induced tissue browning. Store the samples at RT until further processing or imaging. The samples can be stored at 4 °C for long-term storage.

*See General note 13.*

**C. Imaging samples with a two-photon (2P) microscope and a light-sheet microscope (LSM)**

After optical tissue clearing and labeling, samples can be imaged with various microscopes. The samples described in this protocol were both imaged with a two-photon microscope (TPM) and a light-sheet fluorescence microscope (LSFM). Other microscopes, such as confocal laser scanning microscopes (CLSM), are suitable as well. The choice of imaging modality is based on lab infrastructure and research questions, e.g., whether the needed resolution for assessment of structures is in the micrometer range (LSFM) or the submicron range (TPM).

1. TPM

A two-photon laser scanning microscope (Leica TCS SP5 MP, Leica Mikrosysteme Vertrieb GmbH, Wetzlar, Germany), equipped with an HCX APO L 20×/1.00 W water immersion objective, was used. Working distance of the objective was 2 mm, and the excitation source was a 140-fs pulsed Ti:sapphire laser (Chameleon Ultra II, Coherent Inc., Santa Clara, CA, USA), mode-locked at 800 nm, to image the cleared human breast and lymph node samples. Fluorescence emission was detected using photomultiplier tubes (Hamamatsu, R9624, Japan) in three wavelength ranges: 385–489 nm (blue), 489–563 nm (green), and 568–700 nm (red). Imaging settings were optimized for stained structures and Second-Harmonic collagen. Images and image stacks were acquired with Leica Application Suite Advanced Fluorescence (Leica Microsystems). The samples were transferred from the Falcon tube (end of the optical tissue clearing protocol) and imaged in a glass Petri dish, carefully covered with a coverslip and a water drop on the coverslip. This allows for imaging of MASH-cleared samples using a water immersion objective (RI 1.33). Here, shrinkage in thickness was minimal, and final tissue thicknesses ranged from 1 to 3 mm.

2. LSFM

Light-sheet fluorescence microscopic imaging was performed with the Ultramicroscope II (La Vision Biotech, Bielefeld, Germany), equipped with a SuperK Extreme Supercontinuum white light laser (EXW-12, NKT Photonics, Birkerød, Denmark). The objective used was an MVPLAPO 2× C/0.5 NA objective with dipping cap (Olympus, Japan), a working distance of 5.7 mm, and an RI range of 1.33–1.56. The optically cleared samples were fully immersed in ethyl cinnamate (ECi), ranging from 500 to 700 mL, depending on the size of the sample. Eci has been used as the imaging medium to maintain proper refractive index matching (RI 1.56).

## Data analysis

**A. Tissue shrinkage analysis**

In order to analyze the successful clearing and labeling of the breast and lymph node tissue samples, we first analyzed tissue shrinkage. During tissue processing, pictures of samples were taken after every key step, with the samples placed on millimeter paper on a light pad to provide a scale reference. Images were imported into FIJI (ImageJ), and tissue boundaries were manually outlined as regions of interest (ROIs) according to the instructions below.

Step-by-step FIJI instructions:

1. Importing the image: *FIJI* → *File* → *Open* → Select image → *Open*.

2. Setting measurements: *Analyze* → *Set Measurements* → tick *Area* → tick *Area Fraction* → *OK*.

3. Outlining the ROI: *Toolbar* → *Freehand Selection tool* → manually trace around tissue.

4. Measuring ROI: *Analyze* → *Measure*.

5. Exporting results: *Results window → File → Save As → .csv*.

**B. Fluorescence intensity analysis**

To validate the dyes used in our 3D microscopy approach, we measured the fluorescence intensity as a function of the imaging depth. Image stacks were imported into FIJI (ImageJ), where fluorescence intensity measurements were performed within defined ROIs according to the workflow below.

Step-by-step FIJI instructions:

1. Import the image stack: *FIJI → File → Open → Select image stack → Open*.

2. Verify stack dimensions: *Image → Properties* → Confirm that the voxel depth (slice spacing) corresponds to the acquisition settings of the respective image stack → *OK*.

3. Define the ROI: Select the region of interest using the appropriate selection tool.

4. Generate the z-axis profile: *Image → Stacks → Plot Z-axis Profile*.

5. View the profile: A plot window will display the mean fluorescence intensity within the selected ROI as a function of imaging depth.

6. Export numerical data: *Plot window → List → Save As → Save as .csv*.

## Validation of protocol

**Figure 1.**
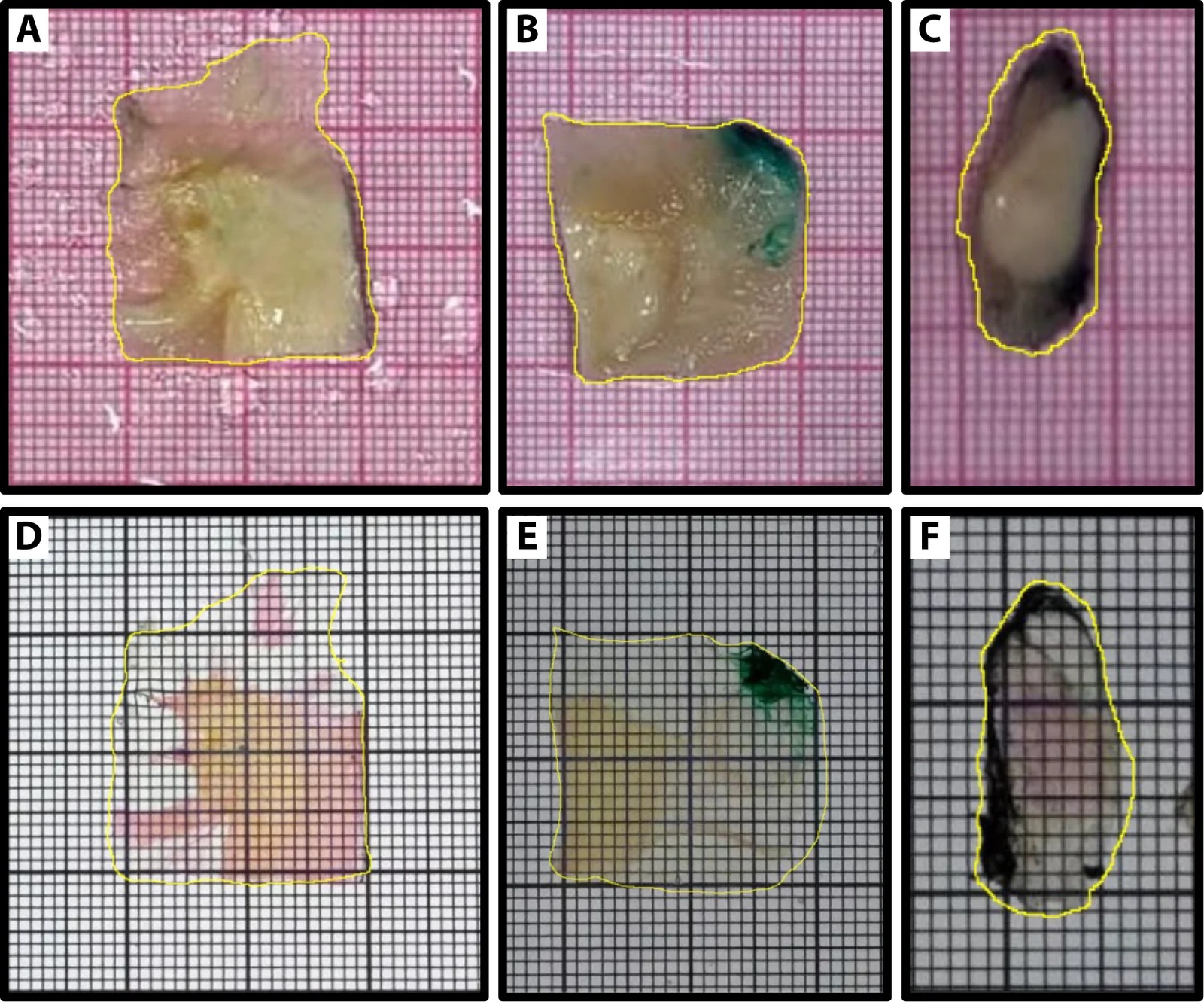
Region of interest (ROI)-based outlining of tissue samples for two-dimensional shrinkage analysis. (A) Breast tissue sample 1, before clearing. (B) Breast tissue sample 1, after clearing. (C) Breast tissue sample 2, before clearing. (D) Breast tissue sample 2, after clearing. (E) Lymph node tissue sample 1, before clearing. (F) Lymph node tissue sample 2, after clearing.

To assess the preservation of tissue morphology, we assessed the tissue deformation that was introduced by the clearing protocol. Tissue shrinkage was evaluated for seven separate tissue samples by comparing the two-dimensional surface area of the samples before and after processing (see detailed steps in the Data analysis section). The tissue area fraction relative to the total image area was measured for all tissue samples (Table 1). Each sample was outlined and measured three times to improve measurement reliability, and the mean value was used for further calculations. Figure 1 illustrates some examples of the ROI outlining used for this analysis. Absolute tissue area was calculated based on the known dimensions of the millimeter paper reference.

**Table 1.** Quantification of tissue area before and after clearing and the resulting tissue shrinkage in breast tissue and lymph node samples

	**Whole image area pre (mm^2^** 2P)	**Mean tissue area fraction pre**	**Mean tissue area pre (mm**P**^2^** P)	**Whole image area post (mm^2^** P)	**Mean tissue area fraction post**	**Mean tissue area post (mm**P**^2^** P)	**Mean shrinkage (mm^2^** P, %)
Sample 1: non-malignant	2,500	0.10852	271.3	1,400	0.19036	266.504	4.796, 1.8
Sample 1: malignant	1,800	0.2579	464.22	1,500	0.30894	463.41	0.81, 0.2
Sample 1: lymph node 1	3,000	0.0282	84.6	1,200	0.06389	76.668	7.932, 9.4
Sample 1: lymph node 2	3,000	0.08606	258.18	1,200	0.18632	224	34.596, 13.4
Sample 2: non-malignant	2,500	0.17402	435.05	1,400	0.29872	418.208	16.842, 3.8
Sample 2: malignant	1,600	0.26252	420.032	1,200	0.32106	385.272	34.76, 8.3
Sample 2: lymph node 1	2,000	0.14778	295.56	1,050	0.256	268.8	26.76, 9.1
Sample 2: lymph node 2	2,000	0.07174	143.48	1,050	0.10368	108.864	34.616, 24.1
Sample 3: malignant	1,600	0.30487	487.792	1,600	0.2858	457.28	30.512, 6.3

Across all seven samples that were used to validate the protocol, the mean tissue surface area decreased from 317.80 ± 135.61 mmP^2^P before processing to 296.51 ± 136.19 mmP^2^P after treatment, corresponding to an average reduction of 21.29 ± 13.88 mmP^2^P, or approximately 6.7% of the original surface area. This level of tissue shrinkage falls within the range reported for comparable optical tissue clearing protocols [3,9,14], suggesting that the protocol preserves overall tissue morphology within acceptable limits. Tissue depths were also approximated visually using millimeter paper; however, due to the imprecision of the method and varying tissue thicknesses across the tissue area, the 3D tissue shrinkage analysis was not performed.

**Figure 2.**
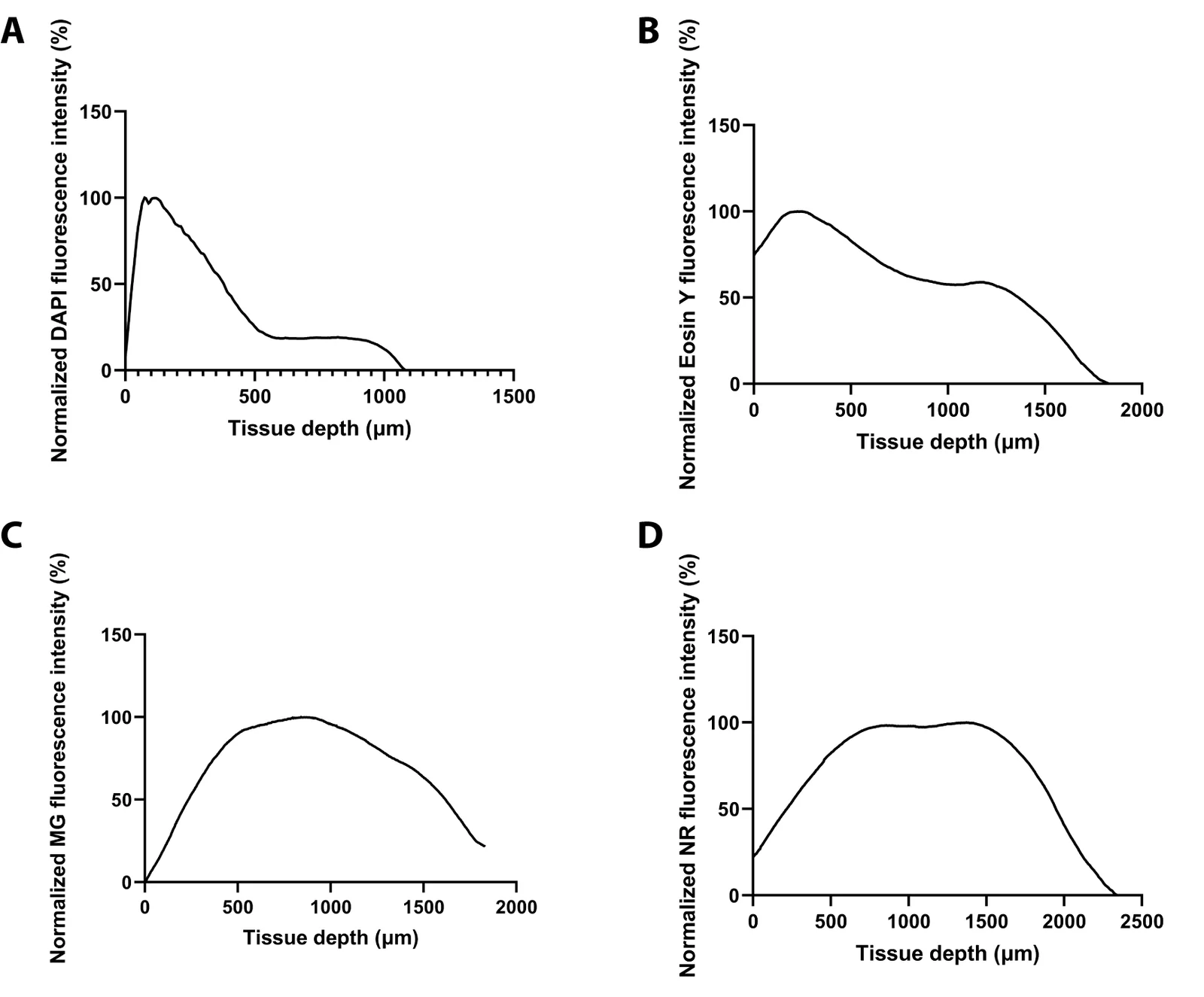
Depth-dependent fluorescence intensity profiles of tissue-clearing dyes and nuclear stains. Normalized fluorescence intensity was measured within defined regions of interest (ROIs) across the imaging depth of cleared breast tissue samples. (A) DAPI fluorescence quantified from two-photon microscopy (TPM) images. (B) Eosin Y fluorescence quantified from light-sheet fluorescence microscopy (LSFM) images. (C) Multiscale architectonic staining of human cortex (MASH)-Methyl Green (MG) fluorescence quantified from LSFM images. (D) MASH–Neutral Red (NR) fluorescence quantified from LSFM images. The profiles illustrate signal distribution and attenuation as a function of imaging tissue depth, ranging from 1,500 to 2,500 μm.

Furthermore, fluorescence intensity was quantified as a function of imaging depth to assess signal distribution and penetration throughout the tissue volume. Image stacks were analyzed in FIJI (ImageJ) according to the steps described in the Data analysis section, and fluorescence intensity measurements were performed within regions where cells were present in the whole field of view. For LSFM datasets, the fluorescence signals of MASH-MG, MASH-NR, and eosin Y were analyzed, while DAPI fluorescence was quantified from TPM images. Mean fluorescence intensity values within the selected ROI were obtained across the z-axis using the *Plot Z-axis Profile* function, generating depth-dependent fluorescence intensity profiles. The resulting fluorescence intensity and depth data were exported to Microsoft Excel, normalized to the maximum intensity of each small molecule dye, and subsequently plotted using GraphPad Prism version 8.2.1 (GraphPad Software, San Diego, CA, USA). These analyses enabled evaluation of signal distribution throughout the tissue thickness, providing insight into dye penetration and depth-dependent signal attenuation. Representative depth-dependent fluorescence intensity profiles are shown in Figure 2.

The fluorescence intensity profiles demonstrated that signal distribution varied across imaging depth and differed between dyes. All profiles showed an initial increase in fluorescence intensity, which may be explained by surface-related artifacts, including tissue edge effects, handling-induced damage, refractive index mismatch at the tissue-medium interface, or incomplete representation of tissue within the selected ROI near the sample surface. The decrease in signal at greater depths is consistent with depth-dependent attenuation caused by light scattering, absorption, shadowing, and reduced fluorescence detection in deeper tissue regions. The different peak positions observed between DAPI, eosin Y, MASH-MG, and MASH-NR likely reflects differences in the spatial distribution of the structures labeled by each dye, as well as the local tissue heterogeneity within the selected ROIs. In addition, the absolute intensity ranges differed substantially between channels, which is expected because fluorescence intensity is affected by fluorophore brightness, dye concentration, staining efficiency, laser power, detector sensitivity, exposure settings, and background signal. Therefore, absolute signal intensities cannot be directly compared between dyes without normalization and standardized acquisition settings. Overall, these profiles indicate that the staining signal was detectable throughout a large portion of the tissue volume, while the decline at larger depths is expected due to the optical limitations of deep-tissue fluorescence imaging.

**Figure 3.**
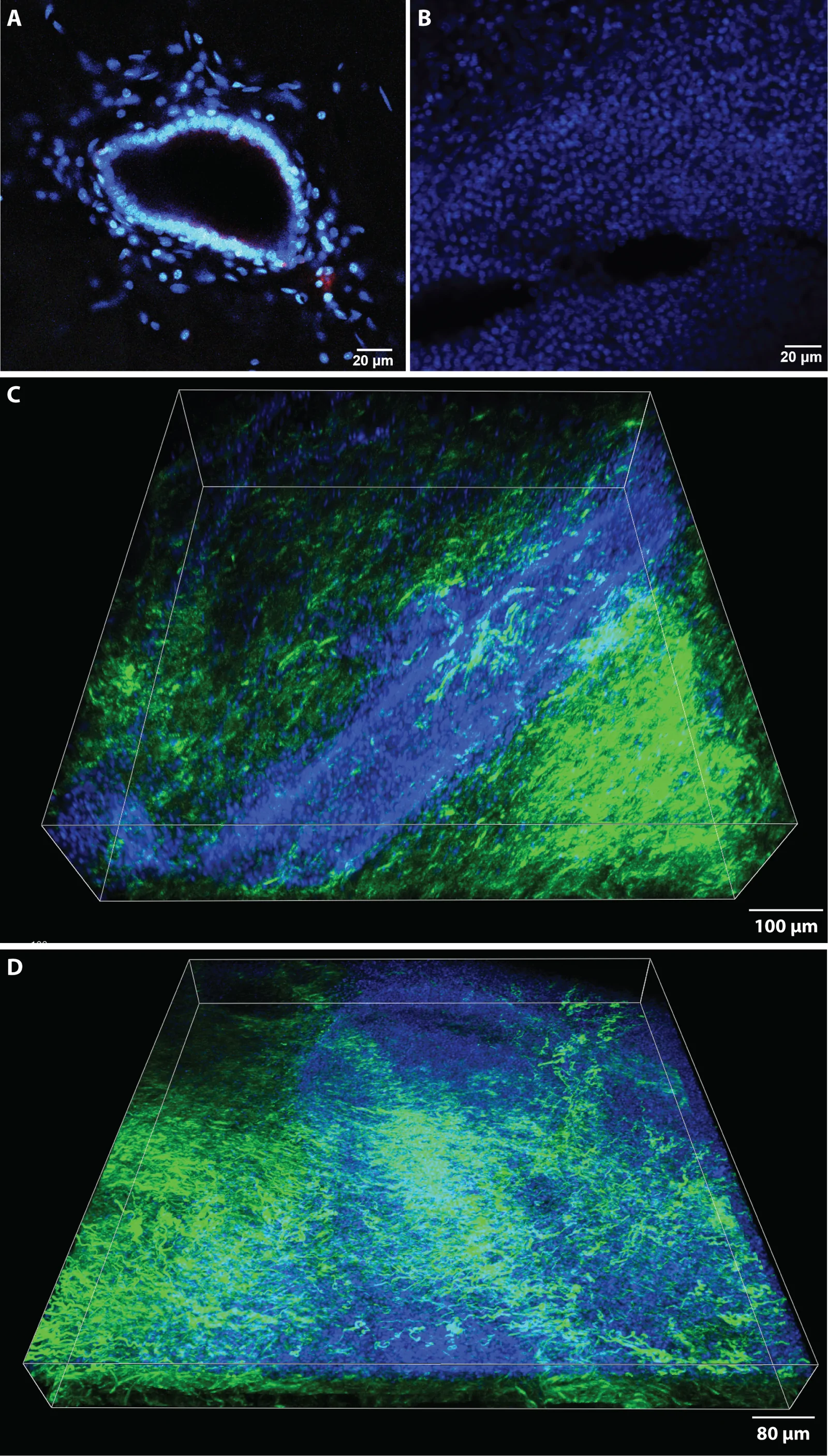
High-resolution two-photon microscopy images after clearing and staining. (A) Vasculature structure of a non-malignant breast tissue and (C) the respective 3D volume. (B) Structural organization of a non-metastatic lymph node tissue and (D) its respective 3D volume. DAPI signal (blue) labels cell nuclei, and the second harmonic generation signal is represented in green (e.g., collagen). Scale bars, 20 µm (A–B), 100 µm (C), and 80 µm (D).

To qualitatively validate the clearing and staining protocol, high-resolution images were acquired using two-photon microscopy. A zoomed-in region and a 3D example of non-malignant breast tissue and an axillary lymph node (without metastasis) are shown in Figure 3.

## General notes and troubleshooting

**General notes**

1. The volumes listed in the Recipes section are based on a total volume of 100 mL, unless otherwise indicated. Required volumes may need to be adjusted depending on the size and number of samples, but are also dependent on the container the sample is in.

2. For Recipe 10 (McIlvaine buffer, pH 4.0), the provided recipe yields 100 mL. The actual volume required will depend on the number of samples processed and the dye(s) selected.

3. In Recipes 11–13, actual dye volumes required will depend on the number of samples being processed. In addition, alternative dyes not covered within the scope of this article may be prepared as needed. Please note that the DAPI staining will not be useful for LSFM, but can be used in TPM.

4. DCM should be handled while wearing two pairs of nitrile gloves. DCM readily permeates many common glove materials, including nitrile, and can rapidly pass through a single glove layer, leading to skin exposure. Double gloving provides limited additional protection by increasing breakthrough time and allowing removal of the outer glove in the event of contamination. If contact with DCM occurs, both glove layers should be removed immediately and replaced with new gloves to minimize dermal exposure.

5. DCM should only be handled and stored in glass containers or compatible fluorinated/polypropylene conical tubes (e.g., Falcon tubes). DCM can dissolve, swell, or extract plasticizers from many common laboratory plastics, which may lead to container deformation, leakage, and sample contamination. Therefore, contact of DCM with non-compatible plastic laboratory equipment should be avoided.

6. Thin tissue sections should be handled with smooth forceps and extreme care, as they are prone to mechanical damage. The usage of a spatula could assist in the careful lifting of the delicate tissues.

7. An average dimension of 30 × 20 × 3 mm for the breast tissues and 20 × 10 × 2 mm for the lymph nodes is assumed for the volumes and incubation times described in the procedure. For larger tissues, different containers are recommended, and the volumes and incubation times have to be adjusted accordingly (see also [15] for MASH-based clearing of larger samples and the required large-scale tissue clearing equipment). The tissues should remain completely submerged at all times.

8. During deparaffinization, incubation times in xylene may vary depending on tissue thickness and fixation. Samples are considered sufficiently deparaffinized once they lose their hard, rigid texture. In some cases, overnight incubation is therefore not required. Prolonged incubation in xylene does not compromise tissue integrity.

9. For temporary storage, samples should be kept in PBS supplemented with thymol to prevent microbial growth.

10. The potassium disulfite solution is reusable and should not be discarded after use. After incubation, the solution can be recovered by filtering through filter paper and returned to the original container.

11. When reusing previously prepared potassium disulfite solution, it should be filtered prior to use to remove any crystals that may have formed during storage.

12. Samples can be incubated with multiple dye working solutions simultaneously. Please note that eosin Y and Neutral Red are not compatible due to the spectral overlap.

13. A similar MASH protocol has been used for brain and prostate samples with larger dimensions, so bigger tissue samples of a different origin could be subjected to this optical tissue clearing and labeling protocol [7,9]. A broader applicability to other FFPE tissues is therefore expected but has to be verified.

**Troubleshooting**

**Table:** 

Problem	Possible cause	Suggested solution
Incomplete deparaffinization	Insufficient xylene incubation time or degraded xylene	Extend xylene incubation steps and use fresh xylene; ensure samples are fully submerged and agitated.
Cloudy Falcon tubes or plasticware	Xylene or DCM dissolve plasticware due to their strong capability of penetrating into polymer chains	Use only glass materials and fluorinated/polypropylene/high-density polyethylene Falcon tubes or plasticware. For other plastics, validate DCM, xylene, and ECi compatibility beforehand.
Tissue becomes brittle and very fragile	Too extensive incubation with organic solvents, as hydrogen peroxide and DCM	Shorten incubation steps with organic solvents.
Tissue remains opaque after clearing	Incomplete dehydration of the tissue or insufficient delipidation	Extend the methanol dehydration times and ensure proper incubation in 100% methanol. You can also go back to re-immersion from ECi into methanol to save the samples. Try repeated washing in 100% methanol. If still opaque when back into ECi, the delipidation was insufficient, and you can go back to the 66% DCM step and delipidate longer. Afterward, wash again in DCM and put in ECi.
Uneven clearing	Different thicknesses within a sample	Ensure proper and even sectioning if possible or pre-cut the FFPE tissue block into a dimension that can be reliably processed.
Uneven staining	Limited penetration in dense regions	Increase permeabilization and labeling time.
Strong autofluorescence during imaging	Incomplete bleaching	Increase time of bleaching step or the concentration of hydrogen peroxide.
Background staining	Insufficient washing after small-molecule labeling	Perform additional washing steps in 60% methanol or ethanol after labeling to extract the dye or reduce dye concentrations.
Tissue damage during rehydration	Methanol gradient is too harsh	Increase incubation times and reduce step size of the methanol gradient.
Tissue swelling or deformation	Abrupt transition between solvents	Make sure to stick to the indicated temperatures; if necessary, increase incubation times and make the solvent exchanges more gradual.
